# Active Suppression of Early Immune Response in Tobacco by the Human Pathogen *Salmonella* Typhimurium

**DOI:** 10.1371/journal.pone.0018855

**Published:** 2011-04-26

**Authors:** Natali Shirron, Sima Yaron

**Affiliations:** Faculty of Biotechnology and Food Engineering, Technion, Israel Institute of Technology, Haifa, Israel; University of Osnabrueck, Germany

## Abstract

The persistence of enteric pathogens on plants has been studied extensively, mainly due to the potential hazard of human pathogens such as *Salmonella enterica* being able to invade and survive in/on plants. Factors involved in the interactions between enteric bacteria and plants have been identified and consequently it was hypothesized that plants may be vectors or alternative hosts for enteric pathogens. To survive, endophytic bacteria have to escape the plant immune systems, which function at different levels through the plant-bacteria interactions. To understand how *S. enterica* survives endophyticaly we conducted a detailed analysis on its ability to elicit or evade the plant immune response. The models of this study were *Nicotiana tabacum* plants and cells suspension exposed to *S. enterica* serovar Typhimurium. The plant immune response was analyzed by looking at tissue damage and by testing oxidative burst and pH changes. It was found that *S.* Typhimurium did not promote disease symptoms in the contaminated plants. Live *S.* Typhimurium did not trigger the production of an oxidative burst and pH changes by the plant cells, while heat killed or chloramphenicol treated *S.* Typhimurium and purified LPS of *Salmonella* were significant elicitors, indicating that *S.* Typhimurium actively suppress the plant response. By looking at the plant response to mutants defective in virulence factors we showed that the suppression depends on secreted factors. Deletion of *invA* reduced the ability of *S.* Typhimurium to suppress oxidative burst and pH changes, indicating that a functional SPI1 TTSS is required for the suppression. This study demonstrates that plant colonization by *S.* Typhimurium is indeed an active process. *S.* Typhimurium utilizes adaptive strategies of altering innate plant perception systems to improve its fitness in the plant habitat. All together these results suggest a complex mechanism for perception of *S.* Typhimurium by plants.

## Introduction

*Salmonella enterica* serovars are enteric bacteria that colonize warm- and cold-blooded animals and are known as causative agents of various human diseases such as typhoid fever, paratyphoid fever and salmonellosis. Most cases of salmonellosis, one of the most frequent food-borne diseases in human, were previously thought to be attributed to consuming contaminated foods originated from animals and particularly poultry products [1]. However recent reports indicate a trend towards fresh produce as a significant source of food-borne illnesses [2]. According to an analysis of outbreaks by the Center for Science in the Public Interest (CSPI), fresh produce is catching up with chicken as a major cause of *Salmonella* infections in the US [3]. In fact, from 2002 to 2003, the percentage of reported outbreaks of *Salmonella* associated with produce in the US was higher than the percentage of poultry related outbreaks [4]. At present *S. enterica* serovars are among the most common causes of food-borne diseases originate from fresh produce, causing about half of the cases [5], [6]. Outbreaks have been linked to a variety of plant products such as fruits, vegetables, sprouts, seeds and leafy greens.

Recent work by food microbiologists and plant pathologists has indicated that the connections between *Salmonella* and plants may be more complicated than simple passive transfer, because the bacteria can attach, survive and even colonize and grow on and in plants [7]–[14]. However the role of the interactions between the host plant and enteric bacteria in control of the colonization on or in the plant is still greatly unknown. In addition, although invasion of *S. enterica* serovars to plants has been reported, the exact conditions that lead to their endophytic survival are not clear. Most observations indicated that *S. enterica* serovars colonize plants without causing disease symptoms [7]–[15], but other investigators have shown that *Salmonella* causes symptoms such as slow growth, wilting and chlorosis in the infected plants [16], [17]. While some reports indicate proliferation of *Salmonella* in the plant [7], [17], in other field studies, the population sizes of *Salmonella* steadily declined after inoculation of the plants. This can be attributed to nutrient limitation, harsh physicochemical conditions (such as temperatures, water activity, osmotic conditions, UV radiation, etc) that fluctuate widely and rapidly over short periods of time, interactions with the plant microflora and plant defense mechanisms [5], [18].

Plants and mammals have fundamental biological differences that affect their capacity to defend themselves against potential pathogenic microorganisms. Unlike mammals that protect themselves by both adaptive immunity and innate immunity, plants rely solely on innate immunity. Mammals have a circulatory system, which is able to deliver immune cells or molecules to sites of infection, while plants lack such kind of circulatory system and they depend on cell-autonomous events. On the other hand, plant cells have a rigid, cellulose-based cell wall that provides an effective barrier to microorganisms. Penetration of the cell wall exposes the host plasma membrane to the microorganisms, where they encounter extracellular surface receptors that recognize pathogen-associated molecular patterns (PAMPs). PAMPs include a growing list of essential conserved microbial molecules that are not normally present in the plants such as lipopolysaccharides (LPS), flagellin, glycoproteins, chitin etc [19]. These PAMPs are detected by extracellular receptors, the pattern recognition receptors (PRRs) which belong to two classes: transmembrane receptor kinases and transmembrane receptor-like proteins. Perception of a microorganism at the cell surface initiates PAMP-triggered immunity (PTI), which usually prevents microbial growth and halts infection before the microbe gains a hold in the plant [20]. PTI may include induction of medium alkalinization [21], production of reactive oxygen species (ROS) such as superoxide and hydrogen peroxide, activation of mitogen-activated protein kinases (MAPKs) [22] and callose deposition to reinforce the cell wall at sites of infection [23]. Successful pathogens are able to escape the plant defense by an active suppression of the PTI response. During infection they are able to multiply in the plants by secretion of effector proteins that interfere directly with PTI responses and alter plant physiology [24]. In gram negative bacteria a type III secretion system (TTSS) deliver these effector proteins directly into the plant cells. Once pathogens acquired the capacity to suppress primary defenses, plant developed more specialized mechanisms to detect and inhibit the evaded microbes: the effector triggered immunity (ETI), in which the plants activate specific plant resistance (R) proteins to inhibit the effector proteins and to suppress microbial growth [20]. Generally, PTI and ETI induce similar responses, but ETI is usually qualitatively stronger and faster and often resulted in hypersensitive response (HR), a localized response which results in a rapid death of the bacteria and the infected plant cells with no spread of bacteria to surrounding tissues. An example is the response of *Nicotiana tabacum* to *Pseudomonades syringe* pv tomato [25]. During the HR the plant cells produce and secret reactive oxygen species (ROS), nitric oxide, Ca^2+^ and H^+^ ions as well as molecular signals like jasmonate (JA), salicylic acid (SA) and ethylene (ET) [26]. In sensitive plants that do not produce the appropriate R proteins the delayed cell response is resulted in proliferation and spread of the bacteria to other parts of the plant. This reaction is induced by pathogenic host bacteria such as *P. syringe* pv tabaci in *N. tabacum* and results in a disease [25], [26]. In other cases no reaction is observed after exposure to the bacteria. An example is the saprophytic bacterium *Pseudomonas fluoresences* [27].

Despite the differences between the nature of the hosts, plant and human pathogens share many pathogenicity determinants like exopolysaccharides (EPS), flagella, pili and toxins suggesting that the plant invasion and colonization process of enteric pathogens may be similar to some phytopathogens. Furthermore, some enzymes and virulence factors play similar roles in animal and plant hosts. For example, aggregative fimbriae have a role in binding and invading epithelial cells in *Escherichia coli* and *S. enterica* [28], and are also implicated in the attachment of these pathogens to alfalfa sprouts [11], [29], [30]. By employing microarray analysis to investigate how *E. coli* O157:H7 adapts to physicochemical conditions in injured lettuce tissue, Brandl et al. revealed the upregulation of numerous genes, some are associated with attachment and virulence in animals or with resistance to oxidative stress [31]. Mutants of the human pathogen *Staphylococcus aureu*s disrupted in genes involved in animal pathogenesis were attenuated in their ability to infect *Arabidopsis thaliana* [32]. Additional examples are the TTSSs, which play a major role in pathogenicity of many Gram-negative plant and animal pathogens (reviewed in [33]). TTSSs are conserved across the plant and animal pathogens, although the injected effectors may differ. The effectors subvert the normal function of the cell or destroy its communication. In contrast to human pathogens, in phytopathens, the exact role of most TTSS-effectors still remains unclear, but in recent years it has been shown that many of them promote disease through suppression of host defense mechanisms [34]. *S. enterica* serovars harbor two distinct TTSSs for virulence proteins as part of being a facultative intracellular pathogen that can survive and replicate intracellularly in mammals [35]. Several effector proteins of *S.* Typhimurium such as AvrA are highly homologous to effector proteins from phytopathogens, anticipating that some of the host-pathogen interactions might also be evident in the interactions with plants [36]. Indeed removing of *Salmonella* TTSS-1 and some effector proteins by deletion of the pathogenicity island 1 (SPI1) affected the bacterial colonization on *Medicago truncatula* [37].

The complex picture of *S. enterica* strains interact specifically with plants is beginning to be elucidated, but still very little is known about the plant perception systems during infection. It was suggested that genetic aspects of both the host plant and *Salmonella* are involved in endophytic colonization [13], [38]. Treating *Medicago sativa* and *Arabidopsis* with ethylene precursor ACC significantly reduced endophytic colonization by *S. enterica* serovar Typhimurium [37]. A recently published work has shown that *S*. Typhimurium triggers the activation of plant immune responses including enhanced transcription of pattern recognition genes [39]. Moreover, JA and ET pathways were found to be of major importance for inducing defense responses during *S*. Typhimurium infection [17].

Taken together, the indication of these specific host-bacteria interactions between *Salmonella* and plants suggests the existence of an important signaling and response crosstalk between plants and *Salmonella* that might play an important role in the persistence of *Salmonella* in plants. To understand how *S*. Typhimurium survives endophyticaly, we studied its ability to elicit or evade the short-term plant response mechanisms of *Nicotiana tabacum* in comparison to the defense response elicited by the plant incompatible pathogen *Pseudomonas syringae* pv. tomato, and identified factors of *S.* Typhimurium that suppress the plant response.

## Materials and Methods

### Plants, plant cell cultures, bacterial strains and culture conditions

Tobacco (*Nicotiana tabacum*) plants were grown in soil in a growth chamber under 14-h-light/10-h-dark (day/night) at 25°C and a relative humidity of 40%.

The *N. tabacum* BY-2 cell line (derived from *N. tabacum* Bright Yellow 2) [40] was maintained by weekly dilution (1∶50) in fresh liquid Murashige and Skoog (MS) media (Sigma, St. Louis, MO) supplemented with 0.2 g L^−1^ KH_2_PO_4_, 1 mg L^−1^ Thiamine, 0.2 mg L^−1^ 2,4-Dichlorophenoxyacetic acid (2,4-D), 30 g L^−1^ sucrose and 0.2 g L^−1^ myoinositol at pH 6.2. Wild type (WT) *Salmonella enterica* serovar Typhimurium (*S.* Typhimurium) strains used in this study are ATCC14028 and SL1344 [36]. All *Salmonella* mutants (Δ*invA*, Δ*rfaH* and Δ*phoP*) were kindly obtained from Dr. Wolfgang Rabsch from Robert Koch-Institute, Germany. The *invA* null mutant carries a deletion in the translocon gene *invA*, rendering it unable to translocate any effector protein of TTSS-1 [41]. RfaH is a transcriptional antiterminator that reduces the polarity of long operons encoding secreted and surface associated cell components of *Salmonella*, including O-antigen and lipopolysacchaide (LPS) core sugars. Mutant Δ*rfaH* is considered as an efficient vaccine against subsequent challenge by WT *Salmonella* in a mouse model [42], [43]. PhoP (together with PhoQ) positively and negatively regulates the production of over 40 proteins involved in virulence. Strains with null mutations in *phoP* attenuates virulence in mice and humans and are unable to survive within macrophages [44]. All *Salmonella* strains were transformed with the pGFP plasmid (Clontech, Palo Alto, Calif.) to obtain Green Fluorescent Protein (GFP)-labelled cells, and were cultured in LB medium with antibiotics, when required. *Pseudomonas fluorescens* 2P24 and *Pseudomonas syringae* pv. tomato DC3000 were grown for 48 h on King's B medium [45]. In the case of *P. syringae* the media was supplemented with 50 µg ml^−1^ rifampicin and in the case of *P. fluorescens* 100 µg ml^−1^ ampicillin was added. All strains were stored as glycerol (20%) cultures at −70°C.

### Infection of plants with bacteria by infiltration

Infections were performed by bacterial infiltration of *N. tabacum* leaves using saline containing bacteria at a density of 7.5 log CFU ml^−1^. Bacteria were harvested by centrifugation (4000 g for 20 min at 4°C) suspended in saline and adjusted to appropriate cell density estimated spectrophotometrically at 600 nm. Infiltrations of the bacterial suspension or saline only as control were carried out by injection without a syringe to the abaxial surface of the leaf as described [46]. All experiments were conducted 4 times in duplicates and the developed symptoms were recorded for 7 days post infiltration.

### Infection of plants with *Salmonella* through plant irrigation

When Tobacco plants reached an average height of 20–30 cm, they were drip irrigated with the contaminated water. Overnight cultures of *S*. Typhimurium (pGFP) were diluted (1∶100) in fresh LB broth supplemented with ampicillin and incubated for 2.5 h at 37°C. Cells were harvested by centrifugation (4000× g for 20 min at 4°C) and re-suspended in 500 ml tap water to yield a final concentration of about 7.5 log CFU ml^−1^. Each planter was manually drip irrigated three days with freshly prepared 100 ml of contaminated water by direct application of the pipettes to the soil at a depth of 1 cm from the surface. In this manner, water was not allowed to come into direct contact with the plants phyllosphere. Control planters were placed in parallel in the same room and were treated in the same way with *Salmonella*-free water. For enumeration of bacteria associated with the plants, leaves of each planter were aseptically harvested and weighed a day after the third irrigation step. Care was taken to collect only the superior leaves (at least 10 cm above the soil) that had not come into any contact with the soil. Samples of 20 g were added to 100 ml saline solution in a sterile stomacher bag and pummelled in a stomacher for 3 min. Serial dilutions (1∶10 in saline) were prepared for plate counting. Colonies of *S.* Typhimurium, which were fluorescent under UV light, were counted as described [47]. Four independent experiments were performed in duplicates and results were statistically analyzed as states below.

### Attachment of *S*. Typhimurium to *N. tabacum* BY-2 cells

Attachment assays were carried out by inoculating 15 ml of cell suspension (five days after sub-culturing) with 8 logs CFU *S*. Typhimurium g cells^−1^. The co-cultures were maintained in 50 ml Erlenmeyer flasks for 1 to 6 h at 25°C on a rotary shaker. Following incubation, cultures were vortexed, centrifuged (1000 g, 3 min, at room temperature), washed three times with PBS and filtered through 20 µm Millipore filters as described [48]. The free bacteria were then collected and their concentration was determined by a viable cell count of the filtrate. In addition the plant cells were lysed by incubation for 10 min at room temperature in lysis buffer (0.2% Triton X-100, 100 mM potassium phosphate buffer, pH 7.8), and the attached bacteria were plate-counted as described [49]. At least 4 independent experiments were performed in duplicates and results were statistically analyzed as states below.

For microscopic analysis, 1 ml of cell suspension (corresponding to approx. 0.1 g) was applied to each well, containing 10^8^ CFU ml^−1^ of GFP-expressing bacteria. Co-cultures were vortexed, washed 3 times with PBS and treated with propidium iodide (70 µg ml^−1^). Aliquots were placed on glass microscope slides and visualized by a fluorescence microscope (Carl Zeiss, Inc) or a Laser Scanning Confocal Microscope (LSCM, Confocal Zeiss LSM 510 META). For the fluorescence microscope, the green fluorescence of pGFP-labelled *Salmonella* was detected using an excitation/emission wavelength of 460/510 nm. The red fluorescence of propidium iodide stained cells was detected using an excitation/emission wavelength of 536/617 nm. In the LSCM, a green argon-ion laser (488 nm) set on 3% power was used for excitation, with 525-nm emission. Images were obtained with the Zeiss LSM image browser software (version 4).

### Measurements of extracellular alkalinization

Induction of medium alkalinization in the plant cell cultures after exposure to the bacteria was measured as described [50]. *N. tabacum* BY2 cell suspension cultures (five days after sub-culturing) were harvested by centrifugation (1500 g, 20 min at room temperature) and resuspended in non-quenching medium (3% w/v sucrose, 10 mM Tris HCl pH 7.5, 0.5 mM CaCl_2_). Aliquots of 150 µl were transferred to the wells of a 96-well multiplates and pyranine (8-hydroxypyrene-1,3,6-trisulfonic acid trisodium salt) (Sigma) was added to a final concentration of 3 µg ml^−1^. Next bacteria (approximately 8.5 log CFU g cells ^−1^) were inoculated into 150 µl of cell suspension (corresponding approximately to 0.015 g fresh weight). Resulting fluorescence from the cell suspension cultures, due to elicitor treatment by the different bacteria, was measured continuously for 20 min at an excitation of 460 nm and emission of 510 nm at 24°C±1°C as described [50], and was plotted against a pyranine fluorescence/pH calibration curve, prepared by measuring the fluorescence of cell suspensions cultures in potassium phosphate buffers, adjusted to a given pH (range 6.0–8.5) by mixing a giving volume of 1 M solutions of KH_2_PO_4_ and K_2_HPO_4_ (according to the Henderson-Hasslbalch equation). Four duplicate experiments were performed.

### H_2_DCF-DA fluorescence assay of oxidative burst reaction products

The production of reactive oxygen species (ROS) by both, infiltrated *N. tabacum* leaves and challenged *N.tabacum* BY-2 cell suspension cultures was measured using 2′,7′–dichlorodihydrofluorescein diacetate (H_2_DCF-DA) (Sigma). This non-polar compound is actively taken up by cells and converted by esterases to H_2_DCF, a non-fluorescent molecule, which is rapidly oxidized to the highly fluorescent DCF by peroxides. DCF is excited at 450 nm and the emitted fluorescence is detectable through a 510 nm filter [51]. In each experiment we triggered the plant cells with *S*. Typhimurium WT and mutants, the pathogenic incompatible bacteria *P. syringae* and the nonpathogenic bacteria *P. fluorescens*. The effect of *Salmonella*'s LPS on plant reaction was tested using purified LPS from *S.* Typhimurium, purified by phenol extraction (Sigma) in a final concentration of 50 ng ml^−1^. In addition, we used heat killed *Salmonella* cells, which were pre treated by boiling in distilled water for 10 min and chloramphenicol treated *Salmonella* which had been exposed to sub-lethal concentrations (10 µg ml^−1^) of chloramphenicol (∼1/2 MIC) for 30 min to inhibit bacterial protein synthesis. Control cells were treated with pure LB.

Using various concentrations of bacteria at stationary phase of growth (7 to 9 log CFU g cells^−1^) and varying amounts of cells (0.1 to 0.3 g), we established a cell ratio according to the point were no further significant increase in ROS production was obtained. The highest ROS production was attained with 8 log CFU g cells^−1^. Thus, all further studies regarding the H_2_DCF-DA assay were performed using this cell ratio. We also repeated the above experiments with bacteria-free *Salmonella* supernatant by centrifugation of the bacterial cultures to pellet the cells (10,000 g for 10 min) and transferring 30 µl of the supernatant to the plant cells suspension. To compare the response of the plant cells to *Salmonella* at different grow stages, *Salmonella* at the logarithmic growth stage was also examined. This was done by dilution (1∶100) of an over night bacteria culture in fresh LB medium and incubation of the culture at 37°C with shaking to reach O.D_600_ of 0.4–0.8. Next, the culture was centrifuged and the pellet was suspended in saline to obtain 8 log CFU g cells ^−1^ in co culture with the cell suspension.

In each experiment *N. tabacum* BY-2 cells were diluted in a non-quenching buffer and aliquots of these suspensions (120 µl) were transferred to the wells of 96-well black plates, to which bacteria were added. Stock solution of H_2_DCF-DA was added immediately to obtain a final concentration of 50 µM. Control cells were diluted in non-quenching medium with no elicitor. The fluorescence resulting from the oxidative burst reaction produced by the cells was measured continuously for 1 h in a microplate fluorometer (Biotek, Synergy HT) at an excitation of 460 nm and emission of 510 nm. The resulting fluorescence was expressed as relative ROS production according to this equation: 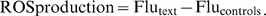


For measurement of ROS production by plant leaves, the first fully expanded leaves were removed from approximately 20 cm (height) plants. Epidermal peels were then removed from the abaxial surface of each leaf and placed into a small Petri dish containing 10 ml of loading buffer (3% w/v sucrose, 10 mM Tris HCl pH 7.5, 0.5 mM CaCl_2_) and 5 µL of H_2_DCF-DA stock solution (100 µM). Peels were loaded in the dark for 10 min, removed and floated on a dish of fresh buffer to wash off excess dye. Individual peels were affixed to a glass cover-slip on which the peel remained immersed in 0.5 ml of loading buffer [52]. For elicitor challenging, bacteria (5 µL of 7 log CFU ml^−1^ in LB medium) were added directly to the buffer. Microscopic examination of tissue ROS production was carried out immediately using the LSCM Zeiss LSM 510 META. A green argon-ion laser (488 nm) set on 3% power was used for excitation, with 525-nm emission. Images were captured before and immediately after elicitor-challenge. Analysis of images was performed using the Zeiss LSM image browser software.

#### Statistical analysis

Results were statistically processed using the One Way Analysis of Variance (ANOVA) method, followed by the Tukey-Kramer test when needed. *P* values ≤0.05 were regarded as significant.

## Results

### Transfer of *S.* Typhimurium from contaminated irrigation water to the leaves and its effect on the plant

Tobacco plants have been extensively used as a model for studying the initial steps of immune response against epiphytes, symbionts and phytopathogens. Thus we decided that the model system of our research will be *S*. Typhimurium and *N. tabacum*. Since information about the persistence of *Salmonella* on tobacco plants was not available in the literature, we investigated whether *S.* Typhimurium transmits from contaminated irrigation water to the phyllosphere of *N. tabacum* plants, if it is able to persist in the plants and if it causes observable changes in the plants. GFP-expressing *S.* Typhimurium was detected in all leaves of the experimental samples following drip irrigation with contaminated water, while no GFP-expressing colonies were observed in the control plants irrigated with tap water. *S.* Typhimurium was detected in leaves at an average concentration of 3.7 (±0.4) log CFU g^−1^ leaves. There was no visible damage to the contaminated plants or their leaves even 10 days after contamination (data not shown).

**Figure 1:**
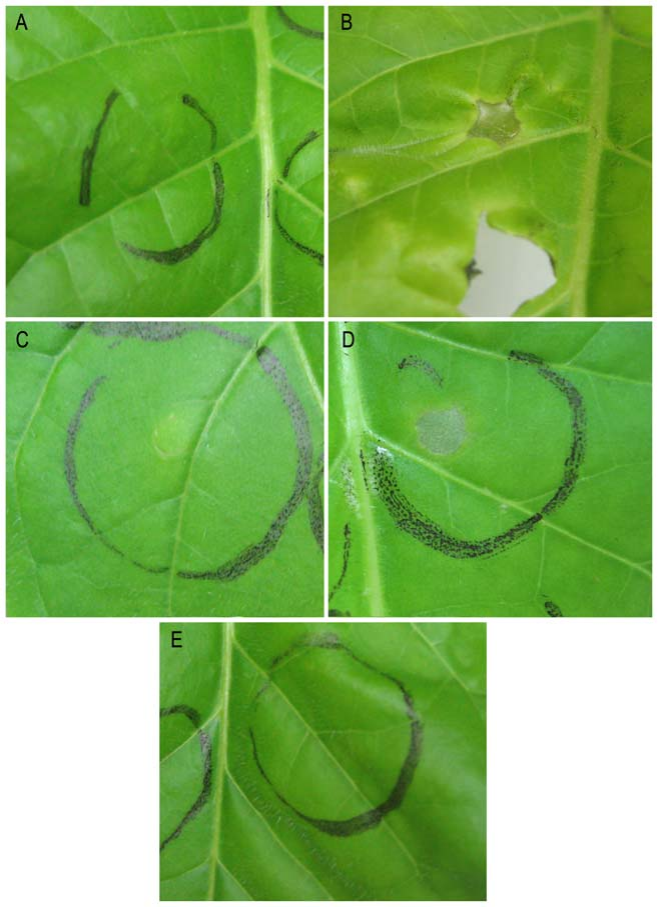
Elicitation of response in *N. tabacum* mature leaves. Leaf panels were infiltrated with approximately 7 log CFU ml^−1^ of (A) *S.* Typhimurium, (B) *P. syringae* pv. tomato, (C) *P. fluorescens*, (D) *S.* Typhimurium *ΔinvA* mutant; and (E) *S.* Typhimurium *ΔrfaH* mutant. Leaf tissue collapse was evident within 24 h in plants infiltrated with *P. syringae* pv. tomato (B), chlorotic primary lesions were induced in resistant leaves by *P. fluorescens* 48 h following plants infiltratation (C). No visible reaction to infiltration of *S.* Typhimurium wt and *ΔrfaH* mutant was observed (A and E). However, necrosis was evident 4 days following infiltration with *Salmonella ΔinvA* mutant (D).

Since the infected *N. tabacum* plants grew well and we did not observe any disease symptoms, we decided to further investigate if *S.* Typhimurium causes symptoms in *N. tabacum* leaves after infiltration. The incompatible plant pathogen *P. syringae* and the saprophytic *P. fluorescens* were used as positive and negative controls, respectively. As expected, *N. tabacum* responded to infection by *P. syringae* with the development of HR- like symptoms (Figure 1B). Necrotic local lesions appeared 1 to 3 days post inoculation (infiltration), and the bacteria infection remained limited to the inoculated leaf. *P. fluorescens* inoculated leaves developed only mild local chlorotic lesions 2 to 7 days post inoculation (Figure 1C). These observations were totally different from leaves infected with *S.* Typhimurium. In the *S.* Typhimurium infected leaves we could not detect any sign of visible response (Figure 1A).

### *S.* Typhimurium elicits low levels of ROS production in *N. tabacum* epidermis

**Figure 2:**
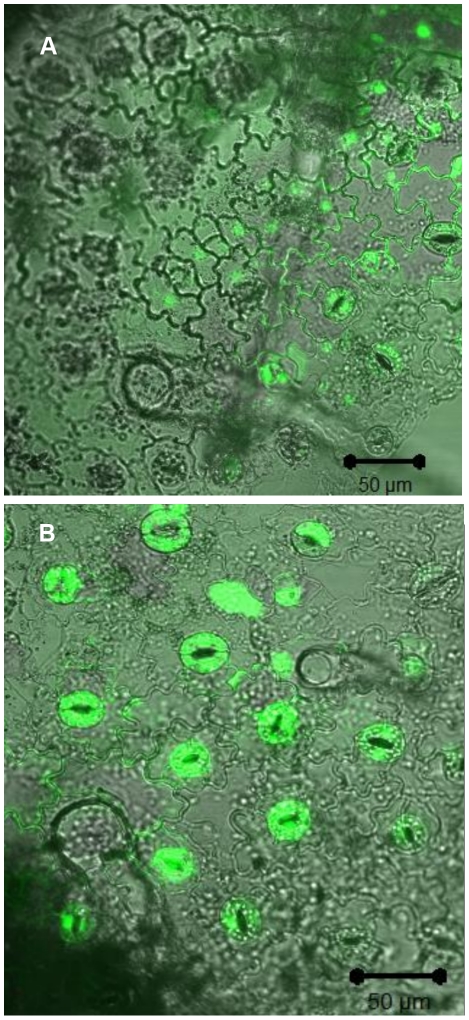
Laser scanning confocal imaging of the elicited- oxidative burst in the outmost epidermis cell layer of *N. tabacum*. Epidermal tissues were loaded with H_2_DCF-DA, washed, and examined by laser scanning confocal microscopy. *S.* Typhimurium (A) and *P. syringae* (B) were added during time course of image acquisition. The images show green fluorescence mainly in the stomata guard cells. Image comparison evaluation demonstrates more than four times fluorescence intensity in image B. The laser setting, microscope filters and all other image parameters were identical in both images.

**Figure 3:**
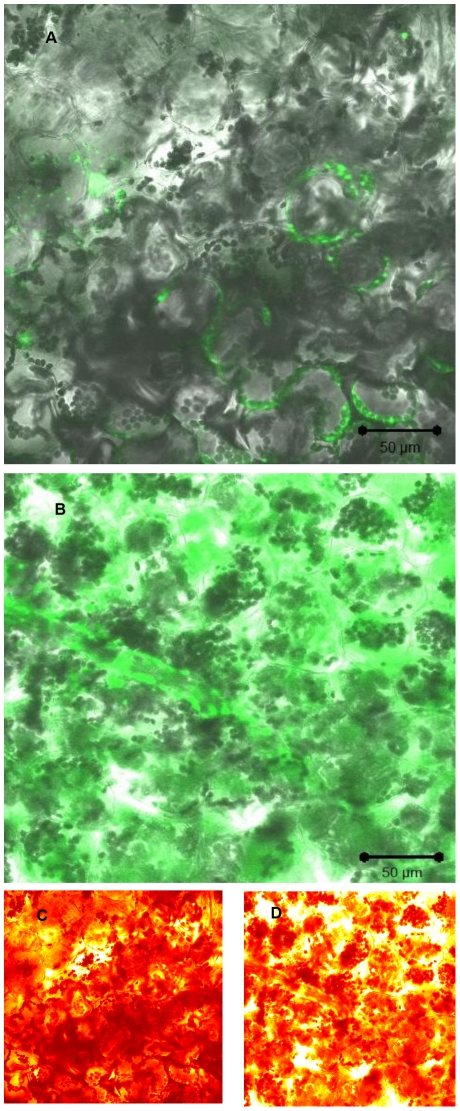
Laser scanning confocal images showing Z stacks projections imaging of infected *N. tabacum* plants showing oxidative burst in parenchyma cells. Plant tissue was loaded with H_2_DCF-DA, washed, and infected with *S.* Typhimurium (A, C) and *P. syringae* (B, D). Epidermal tissues of the plant were examined by laser scanning confocal microscopy. The pseudocolor key was included and was applied to glow scale images (C and D). The laser setting, microscope filters and all other image parameters were identical in both images.

**Figure 4:**
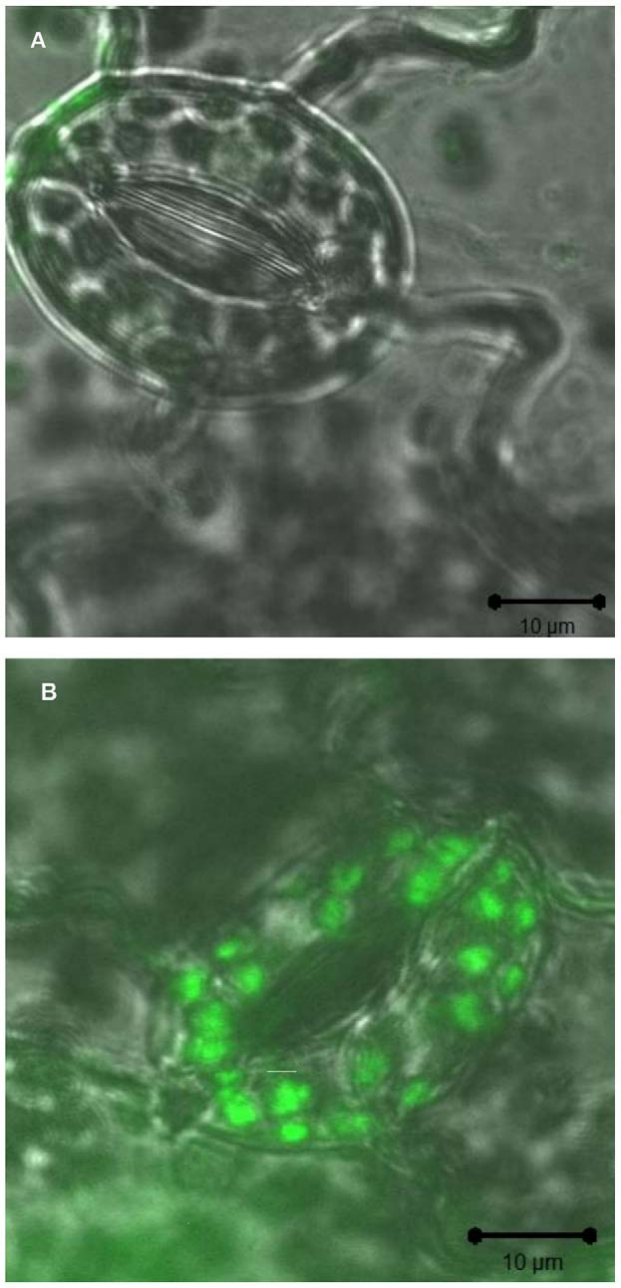
Laser scanning confocal imaging of the elicited- oxidative burst in stomata cells of *N. tabacum*. Epidermal tissue was loaded with H_2_DCF-DA, washed, and examined by LSCM. *S.* Typhimurium (A) and *P. syringae* (B) were added during the time course of image acquisition. The laser setting, microscope filters and all other image parameters were identical in both images.

To investigate whether *S.* Typhimurium elicits oxidative burst production by the epidermal tissues of the leaves we applied the procedure of measuring DCF (the oxidized product of H_2_DCF) fluorescence as a marker for the presence of ROS compounds [53]. *N. tabacum* epidermis were loaded with H_2_DCF-DA and visualized by LSCM. The cellular features of the *Salmonella*-elicited oxidative burst were compared to those observed with *P. syringae*. The response to both *P. syringae* and *S*. Typhimurium was very rapid, but the intensity was different. After exposure to *P. syringae*, a rapid oxidative burst was observed in most epidermal cells within the plant tissue (Figures 2B, 3B and 4B). Fluorescent cells were also observed in tissues which were triggered by *S*. Typhimurium, but their fluorescence intensity was much lower compared to *P. syringae* (compare Figures 2A/3A/4A to 2B/3B/4B, respectively). Cellular distribution of ROS dependent fluorescence was predominating in guard cells and in parenchyma cells and lower fluorescence was observed in epidermis cells (Figures 2, 3). As can be seen in Figure 3 ROS production was mostly extracellular in the case of *S.* Typhimurium. However, triggering the cells with *P. syringae* resulted in a massive production of ROS, which was scattered inside and outside the cells.

### Adhesion of S. Typhimurium to *N. tabacum* cells in suspension

**Figure 5:**
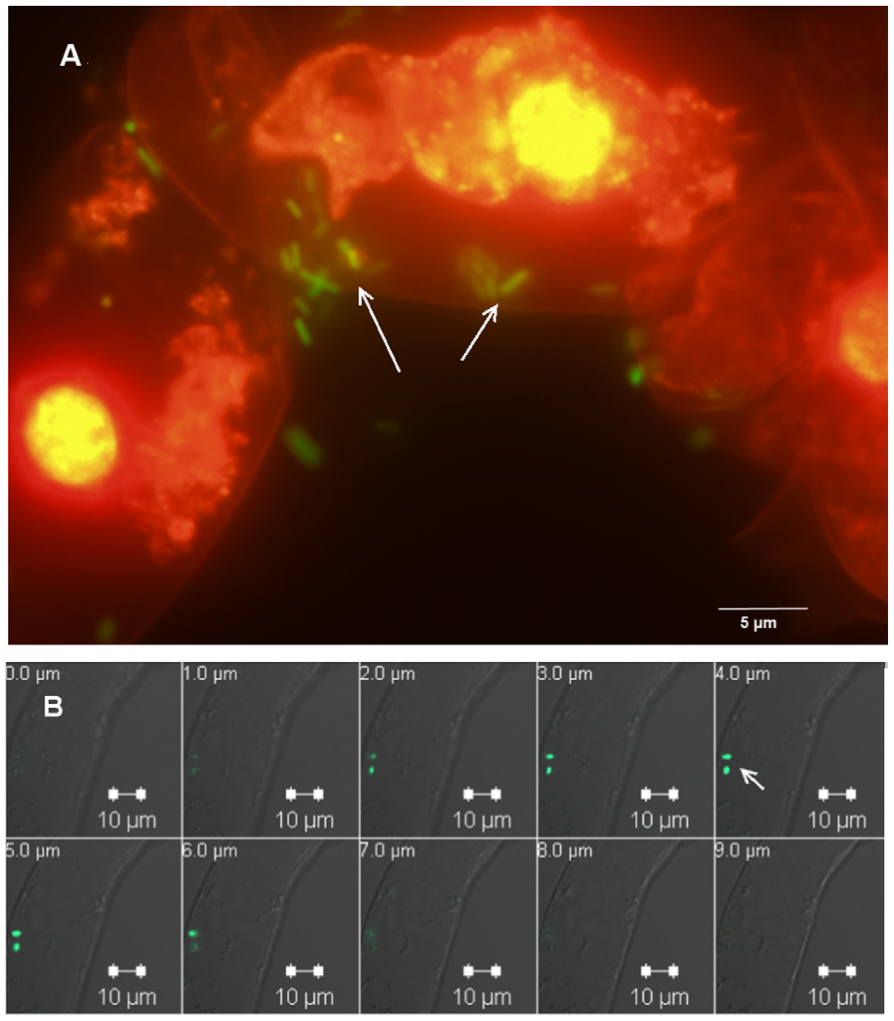
Images of *S*. Typhimurium adhesion to cultures of tobacco BY-2 cells. Cells were grown for 5 days (exponential phase of growth). One ml of cell suspension (corresponding to approx. 0.1 g) was applied to each well, containing 10^8^ CFU ml^−1^ of GFP-expressing bacteria. Cultures were centrifuged, filtered and washed 3 times. Plant cells were treated with propidium iodide (70 µg ml^−1^), that emits a red fluorescence. **A**. Fluorescence microscope images of BY-2 cells with GFP-tagged *S.* Typhimurium (arrows indicate some of the attached bacteria). B. LSCM images of BY-2 cells with GFP-tagged WT *S.* Typhimurium. Optical Z-stacking of the cells allows locating of the bacteria (arrow) at a depth of 4 µm from the surface; few bacteria appear intracellularly.

In order to quantify the plant response to *Salmonella* we looked at the interactions between *S*. Typhimurium and *N. tabacum* BY-2 cell culture. Initial stage was aimed at analyzing the ability of *Salmonella* to adhere to the cells. After 1 h of co-incubation about 50% of the bacteria were found tightly attached to the cells, and after 3 h most bacteria (more than 70%) were attached (about 7.5±0.1 Log CFU g cells^−1^). The attached bacteria were found as single cells or in micro-colonies. Some bacteria bound the cells in the extracellular spaces within the cells clusters (Figure 5). Furthermore, optical Z-stacking suggested that very few bacteria might be located in the intracellular space (Figure 5B).

### The oxidative reaction elicited by different elicitors in *N. tabacum* cell suspension

**Figure 6:**
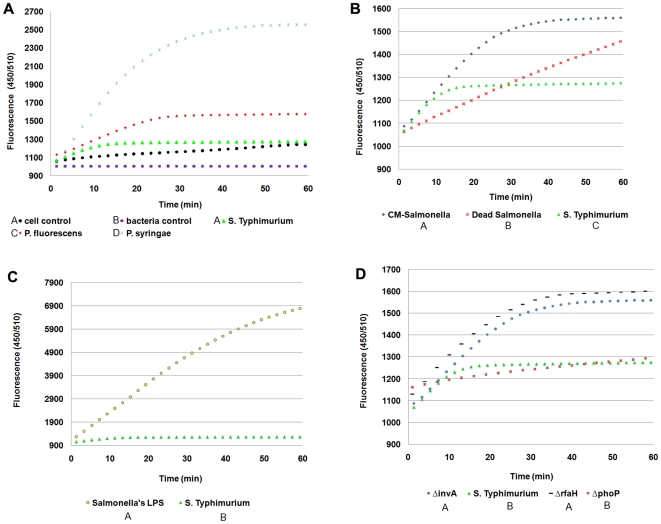
Fluorescence measurements of the H_2_DCF-DA oxidative burst reaction by tobacco BY-2 cells after elicitation. The production of reactive oxygen species (ROS) by tobacco BY-2 cells was determinate by adding of bacteria (8 log CFU g cells ^−1^ in co culture) and measuring the fluorescence resulting from the H_2_DCF-DA (probe for ROS compounds) continuously for 1 h in a microplate fluorometer. (A) Cell suspension was triggered by *S*. Typhimurium; *P. syringae* pv. tomato; *P. fluorescens*, including “cell control” (same experimental conditions no elicitors) and the “bacteria control” (same experimental conditions no cells). *S*. Typhmurium did not trigger an oxidative burst reaction by *N. tabacum* cells. (B) Cells were triggered with heat killed *S*. Typhmurium (Dead *Salmonella*) or chloramphenicol treated *S*. Typhimurium (CM-*Salmonella*). (C) Cells were triggered with purified *Salmonella*'s LPS (50 ng mL^−1^) (“*Salmonella*'s LPS”). (D) Cells were triggered with null mutants of *S*. Typhmurium: Δ*invA,* Δ*rfaH* and Δ*phoP*. *S*. Typhmurium mutants. Δ*invA* and Δ*rfaH* elicitation resulted in a higher level of oxidation than the WT strain and Δ*phoP* mutant. Data in the graphs are mean of 8 results. Results were analyzed by ANOVA followed by a Tukey-Kramer test, for 6 selected points of measurements in the graphs. In each graph, means not followed by the same capital letter are significantly different (*P*<0.05).

Results of the experiments *in-planta* point to the conclusion that *S.* Typhimurium elicits very low levels of reactive oxygen species. To further analyze and quantify the plant response to *S.* Typhimurium we measured the fluorescence of DCF in elicited BY-2 cell suspension [19], [53]. The effect of bacteria challenging on *N. tabacum* cell cultures was initially tested with our positive and negative controls, *P. syringae* and *P. fluorescens*. This was done in parallel with testing the threshold of the self fluorescence of the *N. tabacum* cells (same experimental conditions no elicitors) and the bacteria (same experimental conditions no BY-2 cells). Next, in order to explore the plant reaction to different bacteria, we challenged the cells with various Gram negative and Gram positive bacteria and yeast. All elicitors tested generated an oxidative burst in different scales subsequent incubation. *P. syringae* elicited the highest response - more than 2500 fluorescence units (FU) after 60 min, while *S*. Typhimurium induced the lowest reaction. Fluorescence in elicited BY-2 cells exposed to *Staphylococcus aureus* and *Saccharomyces cerevisiae* was between 2,100 to 2,400 FU, *Bacillus cereus* – 1,830 FU, *Pseudomonas aeruginosa* and *Staphylococcus epidermidis* –1,500 to 1,600 FU, *Serratia marcescens* and *Escherichia coli* - 1,400 to 1,500 FU, and *S*. Typhimurium – about 1,300 FU. In the case of both *S*. Typhimurium strains (ATCC 14028 and SL1344), very low oxidative reaction started a few minutes after elicitation, but was suppressed about 10 min after the initiation of the response (Figure 6A).

Regarding the *S.* Typhimurium depletion response mechanisms, we examined if the induction is contact dependent and if it is influenced by secreted factors of *S.* Typhimurium. For this purpose, we repeated the experiment with bacteria-free *S.* Typhimurium supernatants. Results show no reaction of the cells to the *S.* Typhimurium supernatant (data not shown). To further explore the ability of *Salmonella* to elicit or evade immune response by the plant, we examined the *S.* Typhimurium capability to evade reaction in various growth stages. *S.* Typhimurium in the logarithmic growth promoted a moderate reaction, and levels of oxidative burst were slightly but not significantly higher than the response induced by stationary phase cells (data not shown). When we triggered the cells with purified LPS, heat killed *S.* Typhimurium cells or chloramphenicol treated bacteria an intense reaction was observed in the cells (Figure 6B, 6C). Results differed significantly from live *Salmonella*, however this reaction was not as high as the reaction triggered by *P. syringae*, and its kinetics rate was low. These results show that tobacco cells react to non active *Salmonella* or to LPS of *Salmonella* by producing ROS, a reaction that is inhibited by active *Salmonella* cells.

### Extracellular alkalinization of *N. tabacum* cells suspension

**Figure 7:**
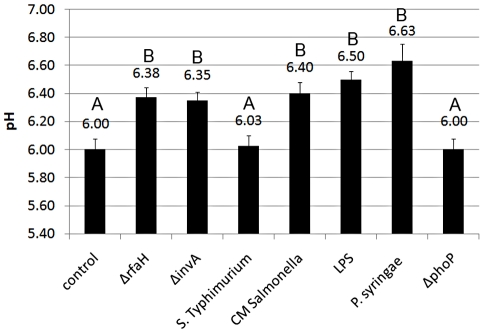
Extracellular pH increase of the culture medium of tobacco BY-2 cells after elicitation. Pyranine (3 µg mL^−1^) was added to the supernatant of the cells and aliquots of these suspensions were transferred to the wells of 96-well plates, to which bacteria (approximately 8 log CFU g cells^−1^) were subsequently added. Resulting fluorescence from the cell suspension cultures was plotted against the pyranine fluorescence/pH curve. *S*. Typhimurium (WT and null mutants Δ*invA* and Δ*rfaH*); *P. syringae*; pure *Salmonella*'s LPS (LPS) and chloramphnicol treated *Salmonella* (CM *Salmonella*). Control cells were treated with the non-quenching medium alone (“control”). Values are pH means of 8 results, and SD values are shown. Means not followed by the same capital letter (A or B) are significantly different (*P*<0.05).

Extracellular alkalinization of the medium is due to ions efflux and influx across the cellular membrane. This sign is among the earliest cellular responses involved in signal perception and signal transduction leading to or associated with the oxidative burst. Changes in pH values of the medium were measured using the pyranine fluorescence assay [50]. The pH before elicitation was constantly monitored to ensure that the observed elicitor-induced alkalinization response was in fact due to elicitor treatment and not other stress factors nor external components. Adding elicitors to the cells suspension resulted in a rapid pH change from the starting pH of 6 to the maximum pH within about 10 min, while control cells, treated with pre-incubation medium alone, showed only very slight variation in pH during the entire experiment. As can be seen in Figure 7, like ROS production, exposure to *S.* Typhimurium resulted in a minor effect on the pH (Not statistically significant), as opposed to *P. syringae* elicitation, which resulted in the most significant pH change (6.6) within 10 minutes after contact (*P*<0.05). Exposure of the cells to bacteria-free *S.* Typhimurium supernatant did not influence the pH (data not shown). Treatment of the *N. tabacum* cell cultures with 50 ng ml^−1^ *S.* Typhimurium LPS resulted in a significant pH increase to 6.5 (*P*<0.05). A similar reaction (6.4) was observed from the elicitation with chloramphenicol treated *Salmonella* (*P*<0.05). These results reveal that LPS of *Salmonella* and non-active *Salmonella* elicit a pH change in tobacco cells, which is inhibited by active *Salmonella* cells.

### The *S.* Typhimurium's TTSS-1 is involved in the inhibition of the plant response

In order to examine the hypothesis of *S.* Typhimurium actively inhibits defense response by the tobacco cells, we triggered the cells and the plants with three null mutants of *S.* Typhimurium. RfaH of *Salmonella* is a regulator of different factors involved in virulence such as the lipopolysaccharide (LPS) core and O-antigen, flagellum/chemotaxis system, TTSS-1 system and SPI4 [42], [43]. When we triggered *N. tabacum* cells with *S.* Typhimurium *ΔrfaH* mutant, ROS production and alkalinization were observed. The rates were higher than *P. fluorescens* but still lower than *P. syringae* (Figures 6, 7). On the other hand this mutant did not show any sign of HR- like symptoms characterized necrosis in infiltrated leaves (Figure 1-E).

InvA is an essential component of the SPI1 TTSS and therefore the *S*. Typhimurium *ΔinvA* mutant is defective in all phenotypes dependent on the TTSS-1 system [54], [55]. When *N. tabacum* cells were triggered with the *ΔinvA* mutant, ROS production and alkalinization were observed, very similar to the response obtained with the *ΔrfaH* mutant (Figures 6D, 7). However, in oppose to *ΔrfaH* mutant, *ΔinvA* mutant induced necrosis in the leaves 4 days following infiltration (Figure 1D).

The PhoP/PhoQ two-component system of *Salmonella* regulates the expression of many effector proteins and other virulent and non-virulant proteins [56]. The response elicited by the *ΔphoP* mutant was very similar to that induced by the WT strain, i.e., no pH changes nor oxidative response (data not shown and Figure 6D).

## Discussion

Enteric bacteria are usually introduced to a new host via contaminated foods or water, colonize its gastrointestinal tract, and are excreted back to the environment through animal feces. Plants can serve as good vehicles for transfer of the pathogen from the environment into the gut of a new host [57]. Thus it is reasonable to propose intimate interactions between the bacteria and the plant to ensure that the enteric pathogens fit the plant environment very quickly and survive in plants long enough and in sufficient numbers for infection of a new host. The study presented here supports this hypothesis by demonstrating that the human pathogen *S.* Typhimurium actively suppresses early stages of defense response in *N. tabacum* plants and cells.

To establish interactions between *Salmonella* and tobacco plants, we initially examined the ability of *S.* Typhimurium to transfer, invade and persist in tobacco plants after irrigation with contaminated water. Although the contaminated water was dripped to the soil without any direct contact with the phyllosphere, the presence of *S.* Typhimurium was confirmed in the leaves and bacteria counts were 3.7 log CFU g^−1^ leaves. In several studies it was shown that different *S. enterica* serovars can be transferred from contaminated water, soil or manure to different plants, and although methods of plants growth and irrigation varied, they usually resulted in similar concentrations of *Salmonella* in the leaves. For instance, in parsley plants that were irrigated with the same *S.* Typhimurium strain 4.1 log CFU g^−1^ leaves were obtained [14], and *S.* Newport reached 2.7 to 3.7 log CFU g^−1^ leaves of lettuce [58]. Like previous observations [7]–[15] *S.* Typhimurium did not cause any visible disease symptoms in tobacco plants, which can imply to plant resistance or bacteria saprophytic nature. In the literature there are only few evidences of *Salmonella* causing disease symptoms in plants. *S.* Dublin reduced the plant biomass and affected the color of the leaves of mini Roman lettuce cv. Tamburo. However these plants were contaminated with more than 6-log CFU g^−1^ leaves [16]. Also, dipping of *Arabidopsis* plants in suspension containing 8 log *S*. Typhimurium ml^−1^, resulted in wilting, chlorosis and eventually death of the infected organs after 2 weeks [17]. These levels are probably far beyond the levels of contamination by enteric pathogens in the field.

Plant response to bacterial elicitation involves early and later reactions aimed at stopping the pathogen invasion into the host tissues, diminishing the proliferation of bacteria and inducing bacterial death. Early reaction towards potential pathogens includes ionic fluxes (primary Ca^2+^, K^+^ and H^+^) and the production of nitric oxide and ROS such as superoxides and hydrogen peroxide [59]. These compounds inhibit bacteria, but together with different additional signaling molecules may also locally activate programmed cell death (PCD), that generates a physical barrier restraining nutrient availability, due to the rapid dehydration caused by tissue death [31], [60]. These defense signaling pathways of plants are not always induced towards pathogens, and each pathogen causes a different reaction in different host [11]. Here the incompatible pathogen *P. syringae* elicited a rapid ROS production in tobacco epidermis almost immediately upon inoculation. As a result, ROS compounds were accumulated in guard cells, in particular within the chloroplast, and in the extracellular spaces in the mezophyll. In contrast to *P. syringae*, *S*. Typhimurium triggered very low levels of ROS production in the epidermal tissue, which was observed by LSCM using the H_2_DCF-DA fluorescence assay, but not by observation of filtrated leaves. In order to evaluate ROS production in a more accurate matter, we tested the kinetics of the ROS accumulation in cell suspension by using the H_2_DCF-DA fluorescence assay. Similarly to the experiments with the whole plants and leaves, *S.* Typhimurium did not promote oxidative burst in the cells as opposed to the incompatible plant pathogen *P. syringae*. Defense signaling pathway on the plant cell surface involves also fluctuation in H+ ions resulting in extracellular pH rise. When we measured the pH changes we got a similar pattern. *P. syringae* elicited a pH raise from 6 to 6.6 while *Salmonella* did not promote any pH change.

One explanation to the phenotype of *Salmonella*'s infected cells is that *Salmonella* might not have been able to bind efficiently to plant cells. In some plant pathogens such as *Agrobacterium tumefaciens* attachment to the host surface is essential for pathogenesis [61], [62]. Image analyses of the *Salmonella* – tobacco co-cultures as well as counting of free and attached bacteria evade this hypothesis, because they indicated that *S*. Typhimurium tightly bound the plant cells and survived during the contact interactions with the host cells. Moreover, few bacteria were even observed inside host cells. We were not able to explain the invasion of the bacteria through the cell wall, because the invaded cells seemed to be undamaged, however, although rare, this observation supports previous experiments with *Arabidopsis* which demonstrated that *Salmonella* has the ability to invade plant cells. In that model system it was suggested that *Salmonella* may even have endopathogenic lifestyle because it proliferated inside the plant cells [17]. In our observations cases of invasion were much less frequent; hence we were unable to determine proliferation in the host cells.

Next, the absence of response of tobacco towards *Salmonella* could have been resulted from a lack of recognition between the attached bacteria and specific surface components of the host that should recognize PAMPs and trigger the signal pathways. However, from previous literature it is already known that *Salmonella* possesses PAMPS that can be recognized by plant cells (e.g. flagellin) [37]. Indeed, when we triggered the cells with *S.* Typhimurium's purified LPS or with heat killed *S.* Typhimurium cells ROS productions as well as alkalinization were observed. A similar observation (low response to living bacteria and strong alkalinization response to boiled bacteria) was described during the analysis of the effect of *Pseudomonas syringae pv tabaci* on cells of tomato, a study that led to the discovery that bacterial flagellins are strong elicitors of defense response in different plants including tobacco [21]. This means that surface components of *S.* Typhimurium elicit the plant response. To confirm that the plant response is not activated by molecules that changed their structure during the heat treatment we repeated the experiment with chloramphenicol treated bacteria. Sub-inhibitory concentrations of chloramphenicol do not kill the cells, but inhibit protein synthesis. Indeed chloramphenicol treated bacteria also elicited the plant response. We can conclude that since only live and active bacteria inhibit response in the cells, components of *Salmonella* have the ability to induce alkalinization and oxidative burst, but the living bacteria actively prevent the cells from reacting to it.

In phytopathogenic bacteria, TTSSs are required for pathogenicity on susceptible hosts, but they are also required for elicitation and/or suppression of plant defense mechanisms [34]. Indeed, when we triggered *N. tabacum* cells with Δ*rfaH* and Δ*invA* null mutants of *Salmonella*, an oxidative burst was generated. The immune attenuated mutant of *S.* Typhimurium Δ*rfaH* down regulates the expression of the TTSS-1 and SPI4 genes and the flagellum/chemotaxis system [42]. InvA is an essential component of the SPI1 TTSS and therefore the *S*. Typhimurium Δ*invA* mutant is defective in all phenotypes dependent on this system, which include bacterial internalization into non-phagocytic cells in mammals and the induction of programmed cell death in macrophages [54], [55]. The levels of LPS and flagellin, potential PAMPs that induce PTI, are not affected by the *invA* mutation and are therefore identical to those of wild type [54]. However it was already shown that TTSS-1 itself or one of its effectors may serve as elicitors in Medicago spp [37]. The similar levels of ROS production and alkalinization triggered by the Δ*rfaH* and Δ*invA* mutants demonstrate that the oxidative burst depletion stimulated by WT *S*. Typhimurium requires a functional TTSS-1. The requirement of a functional TTSS-1 for *S*. Typhimurium to trigger immune response in *N. tabacum* cells suggests the possibility that one or more effector proteins delivered by this system are responsible for the alteration of oxidative burst. However when we triggered the cells with the Δ*phoP* mutant, we did not observe ROS production or pH changes. This means that the mechanism of perception altering does not depend on the regulation of the PhoP/PhoQ two-component system, which regulates the expression of some effector proteins in mammalian cells [56]. However since the conditions in the plant tissue differ from the conditions in the environment of enteric bacteria in the host, it is possible that other regulatory systems control the expression of the effector proteins. Apparently many virulent genes in *Salmonella*, regulated by the PhoP/PhoQ system, are also controlled by other regulatory proteins such as SlyA [63]. When comparing the cells suspension results to the disease symptoms evolved in the leaves after infiltration of the mutants, it can be seen that *S.* Typhimurium WT and its Δ*rfaH* mutant did not promote any observed signs following infiltration to leaves whereas Δ*invA* mutant induced mild local necrosis of the infiltrated area in the leaves. The response of tobacco leaves to the Δ*invA* mutant differs from the response of *medicago* plants to similar mutants of *Salmonella*. In *medicago* plants at least one of the TTSS-1 effectors activate the promoter of PR1, a SA-dependent pathogenesis-related gene, and deletion of these secretion system resulted with hyper colonization [37].

It is concluded that S. Typhimurium actively suppresses some of the pathways in the plant defense response in a mechanism that depends on TTSS-1. At least two mechanisms may explain the suppression of ROS production: (i) secretion of effector proteins which affect the transcription regulation of immunity genes in the plant cells; (ii) secretion of adaptive proteins that protect the bacteria by detoxification of ROS. Both mechanisms are utilized by *Salmonella* to survive in the macrophages [64]–[66], and thus it would be of great interest to determine if they also have a role in suppression of defense response of plants. To our best knowledge this is the first report of an enteric pathogen which acts as a host compatible bacterium of plants in the context of the plant precipitation system. If we extrapolate it further, we can suggest an hypothesis by which similar to plant pathogen that evolved to enhance microbial fitness [26], as *S.* Typhimurium improved its fitness in the plant habitat, it developed an adaptive strategy of altering innate plant perception system. This is not surprising since *S.* Typhimurium can stimulate innate immune responses in cultured epithelial cells through the activity of bacterial effector proteins delivered by its TTSS and in a manner that is independent of innate immune receptors [67]. It should be considered that *Salmonella* is not virulent to plant, but also not avirulant, since some mechanisms of plant response are still triggered by the bacteria. Non-host pathogens developed virulence factors that suppresses or modulate plant defense responses, or help to optimize a hostile environment [68]. We suggest that in the case of enteric pathogens, such as *Salmonella*, some bacterial systems evolved to suppress the immune response in the host also help the pathogen to survive in the plant, although these pathogens are not completely fit to the plant environment. All together these results suggest a complex mechanism for perception of *Salmonella* by *N. tabacum* plants. A better understanding of the mechanisms of plant defense to *Salmonella* will contribute to developing efficient agricultural practices and interventions to avoid or reduce contamination, but will also contribute to our understanding of signaling pathways responsible for host defense in plants.

## References

[1] Tauxe RV (1997). Emerging foodborne diseases: an evolving public health challenge.. Emerg Infect Dis.

[2] Anonymous (2008). http://whqlibdocwhoint/publications/2008/9789241563789_engpdf.

[3] Anonymous (2005). Salmonella Outbreaks Linked to Produce on the Rise, CSPI newsroom.. http://wwwcspinetorg/new/200511211html.

[4] Anonymous (2008). CDC, Centers for Disease Control and Prevention. Annual listing of foodborne disease outbreaks.. http://wwwcdcgov/foodborneoutbreaks/outbreak_datahtm.

[5] Brandl MT (2006). Fitness of human enteric pathogens on plants and implications for food safety.. Annu Rev Phytopathol.

[6] Sivapalasingam S, Friedman CR, Cohen L, Tauxe RV (2004). Fresh produce: a growing cause of outbreaks of foodborne illness in the United States, 1973 through 1997.. J Food Prot.

[7] Gandhi M, Golding S, Yaron S, Matthews KR (2001). Use of green fluorescent protein expressing Salmonella Stanley to investigate survival, spatial location, and control on alfalfa sprouts.. J Food Prot.

[8] Charkowski AO, Barak JD, Sarreal CZ, Mandrell RE (2002). Differences in growth of Salmonella enterica and Escherichia coli O157:H7 on alfalfa sprouts.. Appl Environ Microbiol.

[9] Ercolani GL (1976). Bacteriological quality assessment of fresh marketed lettuce and fennel.. Appl Environ Microbiol.

[10] Islam M, Morgan J, Doyle MP, Phatak SC, Millner P (2004). Fate of Salmonella enterica Serovar Typhimurium on Carrots and Radishes Grown in Fields Treated with Contaminated Manure Composts or Irrigation Water.. Appl Environ Microbiol.

[11] Barak JD, Gorski L, Naraghi-Arani P, Charkowski AO (2005). Salmonella enterica virulence genes are required for bacterial attachment to plant tissue.. Appl Environ Microbiol.

[12] Franz E, Semenov AV, van Bruggen AH (2008). Modelling the contamination of lettuce with Escherichia coli O157:H7 from manure-amended soil and the effect of intervention strategies.. J Appl Microbiol.

[13] Klerks MM, Franz E, van Gent-Pelzer M, Zijlstra C, van Bruggen AH (2007). Differential interaction of Salmonella enterica serovars with lettuce cultivars and plant-microbe factors influencing the colonization efficiency.. ISME J.

[14] Lapidot A, Yaron S (2009). Transfer of Salmonella enterica serovar Typhimurium from contaminated irrigation water to parsley is dependent on curli and cellulose, the biofilm matrix components.. J Food Prot.

[15] Noel JT, Arrach N, Alagely A, McClelland M, Teplitski M (2010). Specific Responses of Salmonella enterica to Tomato Varieties and Fruit Ripeness Identified by In Vivo Expression Technology.. PLoS One.

[16] Klerks MM, van Gent-Pelzer M, Franz E, Zijlstra C, van Bruggen AH (2007). Physiological and Molecular Responses of Lactuca sativa to Colonization by Salmonella enterica Serovar Dublin.. Appl Environ Microbiol.

[17] Schikora A, Carreri A, Charpentier E, Hirt H (2008). The dark side of the salad: Salmonella typhimurium overcomes the innate immune response of Arabidopsis thaliana and shows an endopathogenic lifestyle.. PLoS One.

[18] Brandl MT, Mandrell RE (2002). Fitness of Salmonella enterica serovar Thompson in the cilantro phyllosphere.. Appl Environ Microbiol.

[19] Abulencia A, Acosta D, Adelman J, Affolder T, Akimoto T (2006). Evidence for the exclusive decay B-C(+/-) -> J/psi pi(+/-) and measurement of the mass of the B-C(+/-) meson.. Phys Rev Lett.

[20] Chisholm ST, Coaker G, Day B, Staskawicz BJ (2006). Host-microbe interactions: shaping the evolution of the plant immune response.. Cell.

[21] Felix G, Duran JD, Volko S, Boller T (1999). Plants have a sensitive perception system for the most conserved domain of bacterial flagellin.. Plant J.

[22] Gomez-Gomez L, Felix G, Boller T (1999). A single locus determines sensitivity to bacterial flagellin in Arabidopsis thaliana.. Plant J.

[23] Nurnberger T, Brunner F, Kemmerling B, Piater L (2004). Innate immunity in plants and animals: striking similarities and obvious differences.. Immunol Rev.

[24] Zhou JM, Chai J (2008). Plant pathogenic bacterial type III effectors subdue host responses.. Curr Opin Microbiol.

[25] Huang HC, Schuurink R, Denny TP, Atkinson MM, Baker CJ (1988). Molecular cloning of a Pseudomonas syringae pv. syringae gene cluster that enables Pseudomonas fluorescens to elicit the hypersensitive response in tobacco plants.. J Bacteriol.

[26] Jones JD, Dangl JL (2006). The plant immune system.. Nature.

[27] Sigee DC (1993). Bacterial plant pathology..

[28] Sukupolvi S, Lorenz RG, Gordon JI, Bian Z, Pfeifer JD (1997). Expression of thin aggregative fimbriae promotes interaction of Salmonella typhimurium SR-11 with mouse small intestinal epithelial cells.. Infect Immun.

[29] Torres MA, Jones JD, Dangl JL (2005). Pathogen-induced, NADPH oxidase-derived reactive oxygen intermediates suppress spread of cell death in Arabidopsis thaliana.. Nat Genet.

[30] Lapidot A, Romling U, Yaron S (2006). Biofilm formation and the survival of Salmonella Typhimurium on parsley..

[31] Kyle JL, Parker CT, Goudeau D, Brandl MT (2010). Transcriptome analysis of Escherichia coli O157:H7 exposed to lysates of lettuce leaves.. Appl Environ Microbiol.

[32] Prithiviraj B, Bais HP, Jha AK, Vivanco JM (2005). Staphylococcus aureus pathogenicity on Arabidopsis thaliana is mediated either by a direct effect of salicylic acid on the pathogen or by SA-dependent, NPR1-independent host responses.. Plant J.

[33] Cornelis GR, Van Gijsegem F (2000). Assembly and function of type III secretory systems.. Annu Rev Microbiol.

[34] Mudgett MB (2005). New insights to the function of phytopathogenic bacterial type III effectors in plants.. Annu Rev Plant Biol.

[35] Hansen-Wester I, Hensel M (2001). Salmonella pathogenicity islands encoding type III secretion systems.. Microbes Infect.

[36] Hardt WD, Galan JE (1997). A secreted Salmonella protein with homology to an avirulence determinant of plant pathogenic bacteria.. Proc Natl Acad Sci U S A.

[37] Iniguez AL, Dong Y, Carter HD, Ahmer BM, Stone JM (2005). Regulation of enteric endophytic bacterial colonization by plant defenses.. Mol Plant Microbe Interact.

[38] Dong Y, Iniguez AL, Ahmer BMM, Triplett EW (2003). Kinetics and Strain Specificity of Rhizosphere and Endophytic Colonization by Enteric Bacteria on Seedlings of Medicago sativa and Medicago truncatula.. Appl Environ Microbiol.

[39] Tschape H, Prager R, Streckel W, Fruth A, Tietze E (1995). Verotoxinogenic Citrobacter freundii associated with severe gastroenteritis and cases of haemolytic uraemic syndrome in a nursery school: green butter as the infection source.. Epidemiol Infect.

[40] David KM, Perrot-Rechenmann C (2001). Characterization of a tobacco Bright Yellow 2 cell line expressing the tetracycline repressor at a high level for strict regulation of transgene expression.. Plant Physiol.

[41] Galan JE, Curtiss R (1991). Distribution of the invA, -B, -C, and -D genes of Salmonella typhimurium among other Salmonella serovars: invA mutants of Salmonella typhi are deficient for entry into mammalian cells.. Infect Immun.

[42] Nagy G, Dobrindt U, Hacker J, Emody L (2004). Oral immunization with an rfaH mutant elicits protection against salmonellosis in mice.. Infect Immun.

[43] Nagy G, Danino V, Dobrindt U, Pallen M, Chaudhuri R (2006). Down-regulation of key virulence factors makes the Salmonella enterica serovar Typhimurium rfaH mutant a promising live-attenuated vaccine candidate.. Infect Immun.

[44] Gunn JS, Miller SI (1996). PhoP-PhoQ activates transcription of pmrAB, encoding a two-component regulatory system involved in Salmonella typhimurium antimicrobial peptide resistance.. J Bacteriol.

[45] King EO, Ward MK, Raney DE (1954). Two simple media for the demonstration of pyocyanin and fluorescin.. J Lab Clin Med.

[46] Baker CJ, Atkinson MM, Collmer A (1987). Concurrent loss in Tn5 mutants of Pseudomonas syringae pv. syringae of the ability to induce the hypersensitive response and host plasma membrane K+/H+ exchange in tobacco.. Phytopathology.

[47] Lapidot A, Romling U, Yaron S (2006). Biofilm formation and the survival of Salmonella Typhimurium on parsley.. Int J Food Microbiol.

[48] Marenda M, Brito B, Callard D, Genin S, Barberis P (1998). PrhA controls a novel regulatory pathway required for the specific induction of Ralstonia solanacearum hrp genes in the presence of plant cells.. Mol Microbiol.

[49] Chaboute ME, Clement B, Philipps G (2002). S phase and meristem-specific expression of the tobacco RNR1b gene is mediated by an E2F element located in the 5′ leader sequence.. J Biol Chem.

[50] Agostiano A, Mavelli F, Milano F, Giotta L, Trotta M (2004). pH-sensitive fluorescent dye as probe for proton uptake in photosynthetic reaction centers.. Bioelectrochemistry.

[51] Zeidler D, Zahringer U, Gerber I, Dubery I, Hartung T (2004). Innate immunity in Arabidopsis thaliana: lipopolysaccharides activate nitric oxide synthase (NOS) and induce defense genes.. Proc Natl Acad Sci U S A.

[52] Allan AC, Lapidot M, Culver JN, Fluhr R (2001). An early tobacco mosaic virus-induced oxidative burst in tobacco indicates extracellular perception of the virus coat protein.. Plant Physiol.

[53] Gerber IB, Dubery IA (2003). Fluorescence microplate assay for the detection of oxidative burst products in tobacco cell suspensions using 2′,7′-dichlorofluorescein.. Methods Cell Sci.

[54] Galan JE, Ginocchio C, Costeas P (1992). Molecular and functional characterization of the Salmonella invasion gene invA: homology of InvA to members of a new protein family.. J Bacteriol.

[55] Galan JE, Wolf-Watz H (2006). Protein delivery into eukaryotic cells by type III secretion machines.. Nature.

[56] Groisman EA (2001). The pleiotropic two-component regulatory system PhoP-PhoQ.. J Bacteriol.

[57] Kutter S, Hartmann A, Schmid M (2006). Colonization of barley (Hordeum vulgare) with Salmonella enterica and Listeria spp.. FEMS Microbiol Ecol.

[58] Bernstein N, Sela S, Neder-Lavon S (2007). Assessment of contamination potential of lettuce by Salmonella enterica serovar Newport added to the plant growing medium.. J Food Prot.

[59] Dorey S, Kopp M, Geoffroy P, Fritig B, Kauffmann S (1999). Hydrogen Peroxide from the Oxidative Burst Is Neither Necessary Nor Sufficient for Hypersensitive Cell Death Induction, Phenylalanine Ammonia Lyase Stimulation, Salicylic Acid Accumulation, or Scopoletin Consumption in Cultured Tobacco Cells Treated with Elicitin.. Plant Physiol.

[60] de Pinto MC, Tommasi F, De Gara L (2002). Changes in the antioxidant systems as part of the signaling pathway responsible for the programmed cell death activated by nitric oxide and reactive oxygen species in tobacco Bright-Yellow 2 cells.. Plant Physiol.

[61] Sequeira L (1985). Surface components involved in bacterial pathogen-plant host recognition.. J Cell Sci.

[62] Thomashow MF, Karlinsey JE, Marks JR, Hurlbert RE (1987). Identification of a new virulence locus in Agrobacterium tumefaciens that affects polysaccharide composition and plant cell attachment.. J Bacteriol.

[63] Navarre WW, Halsey TA, Walthers D, Frye J, McClelland M (2005). Co-regulation of Salmonella enterica genes required for virulence and resistance to antimicrobial peptides by SlyA and PhoP/PhoQ.. Mol Microbiol.

[64] Donne E, Pasmans F, Boyen F, Van Immerseel F, Adriaensen C (2005). Survival of Salmonella serovar Typhimurium inside porcine monocytes is associated with complement binding and suppression of the production of reactive oxygen species.. Vet Microbiol.

[65] Mantena RK, Wijburg OL, Vindurampulle C, Bennett-Wood VR, Walduck A (2008). Reactive oxygen species are the major antibacterials against Salmonella Typhimurium purine auxotrophs in the phagosome of RAW 264.7 cells.. Cell Microbiol.

[66] Macvanin M, Hughes D Assays of sensitivity of antibiotic-resistant bacteria to hydrogen peroxide and measurement of catalase activity.. Methods Mol Biol.

[67] Bruno VM, Hannemann S, Lara-Tejero M, Flavell RA, Kleinstein SH (2009). Salmonella Typhimurium type III secretion effectors stimulate innate immune responses in cultured epithelial cells.. PLoS Pathog.

[68] Krzymowska M, Konopka-Postupolska D, Sobczak M, Macioszek V, Ellis BE (2007). Infection of tobacco with different Pseudomonas syringae pathovars leads to distinct morphotypes of programmed cell death.. Plant J.

